# Isolation, Identification, and Investigation of Pathogenic Bacteria From Common Carp (*Cyprinus carpio*) Naturally Infected With *Plesiomonas shigelloides*

**DOI:** 10.3389/fimmu.2022.872896

**Published:** 2022-06-30

**Authors:** Huijie Chen, Yuanli Zhao, Kuangxin Chen, Yulai Wei, Hongrui Luo, Yongming Li, Fei Liu, Zuoyan Zhu, Wei Hu, Daji Luo

**Affiliations:** ^1^State Key Laboratory of Freshwater Ecology and Biotechnology, Hubei Hongshan Laboratory, Institute of Hydrobiology, Innovation Academy for Seed Design, Chinese Academy of Sciences, Wuhan, China; ^2^College of Fisheries, Huazhong Agricultural University, Wuhan, China; ^3^University of Chinese Academy of Sciences, Beijing, China

**Keywords:** natural infection, bacteria identification, pathogenicity, histopathological analysis, tissue bacterial load, comparative transcriptome

## Abstract

Various bacterial diseases have caused great economic losses to the high-density and intensive aquaculture industry; however, the pathogenic mechanism underlying the large-scale challenged to caused by many bacteria remain unclear, making the prevention and treatment of these diseases difficult. In the present study, we isolated a bacterial strain from *Cyprinus* *carpio* having a typical bacterial disease and named it Cc2021. Through subsequent morphological observations, a regression challenge, biochemical identification, and 16S rRNA gene sequence analysis, we determined Cc2021 to be *Plesiomonas shigelloides*. Subsequently, we comprehensively investigated the pathogenicity of *P. shigelloides* in *C. carpio* through a regression challenge and assessed the underlying the pathogenic mechanism. Mortality results revealed that *P. shigelloides* is highly pathogenic and infects various tissues throughout the body, resulting in edema of the liver, spleen, and body and head kidneys. Histopathological analysis revealed obvious inflammation, bleeding, and necrosis in the intestine, spleen, and head kidney. The body’s immune tissues actively produce complement C3, superoxide dismutase, and lysozyme after a challenge to resist bacterial invasion. With regard to the underlying pathogenesis of *P. shigelloides*, comparative transcriptome analysis revealed 876 upregulated genes and 828 downregulated genes in the intestine of *C. carpio* after the challenge. Analysis of differentially expressed unigenes revealed the involvement of major immune pathways, particularly the TNF signaling pathway, interleukin (IL)-17 signaling pathway, and Toll-like receptor signaling pathway. The present study provides new valuable information on the immune system and defense mechanisms of *P. shigelloides*.

## Highlights

1. *P. shigelloides* was isolated and identified from *C. carpio*.
2. *P. shigelloides* challenge led to tissue edema, necrosis, and death in *C. carpio*.
3. The TNF signaling pathway in the intestinal tract of *C. carpio* was induced by *P. shigelloides* challenge.

## Introduction

*Cyprinus carpio* is a valuable and unique fish resource that belongs to the order Cypriniformes and family Cyprinidae (1). As a dominant cyprinid species, *C. carpio* has been reported to account for almost 10% of the annual freshwater aquaculture production (1, 2). However, with the deterioration of the natural environment and the increase in breeding density, the breeding environment of *C. carpio* has been destroyed. In particular, the increasing number of bacterial diseases has had a significant impact on the breeding industry of *C. carpio* and caused huge economic losses (3, 4). The prevention and control of bacterial diseases are areas that need to be urgently addressed for the healthy development of the *C. carpio* industry (5). *Plesiomonas shigelloides*, a member of the Vibrionaceae family, is found in the intestinal tracts of fishes, mammals, and humans (6–8). *P. shigelloides* is widely distributed in the aquatic environment and is considered a new pathogen that causes digestive tract inflammation in many aquatic animals (8). *P. shigelloides* can infect *Trionyx sinensis*, *Takifugu obscurus*, and other aquatic animals alone or in combination; this has resulted in great economic losses to the aquatic aquaculture industry and posed a certain safety hazard to consumers (9, 10). In recent years, *P. shigelloides* has been isolated and identified from several aquatic animals, such as *Percocypris pingi*, *Lota*, *Mylopharyngodon piceus*, and *Carassius auratus* (11–13); however, the pathogenicity of *P. shigelloides* in *C. carpio* remains unknown.

In humans, *P. shigelloides* has been associated with secondary challenges in immunocompromised states, including malignancy, blood disorders, and hepatobiliary diseases (14, 15). In addition to infecting humans, *P. shigelloides* can be pathogenic to aquatic animals, such as *Percocypris pingi* (Tchang), *Huso huso♀ × Acipenser ruthenus♂*, *Takifugu obscurus*, *Ctenopharyngodon idella, and Pelteobagrus fulvidraco*; it has been reported to be infect these species and even cause death (16, 17). The survival of aquatic animals as natural hosts are not affected under normal circumstances (18). The pathogenic bacteria obtained from *Eriocheir sinensis* H. milne Edwards were not found to be the cause of disease in a previous study (19). Moreover, symptoms were found to differ between cold-water fish and warm-water fish (20). Therefore, it can be said that *P. shigelloides* is an opportunistic pathogen that acts once the environment is suitable and has a high fatality rate after onset. After *Ictalurus punctatus* challenge, the mortality rate was found to be up to 60%–100% in a previous study (21). The median lethal dose for Nile tilapia was 1. 425 × 10^8^ CFU/mL, while that for sturgeon was 1.0 × 10^5.8^ CFU/mL (21, 22). *P. shigelloides* is increasingly causing damages to aquaculture; however, its pathogenic mechanism remains unknown. At present, there are many studies on the immunity of carp after a bacterial challenge; the commonly used effective methods are transcriptome sequencing and gene function study. However, few studies have assessed the similarities and differences in immune responses in *C. carpio* after a bacterial challenge based on transcriptome analysis.

Comparative transcriptome analysis has been widely used in immunological studies of different fishes, providing reliable data on immune mechanisms (23). The potential mechanism of antibacterial immunity and the candidate genes has been identified using comparative transcriptome analysis in *Epinephelus lanceolatus* (24). Transcriptome analysis of intestinal epithelial cells of *I. punctatus* has revealed that the key to the invasion of *Edwardsiella ictaluri* is the actin bone in the intestinal barrier (25). In addition to adult tissues and organs, the transcriptome has been used to assess the immune response of fish embryos and primary cells. The innate host immune response of zebrafish embryos infected with *Salmonella* Typhi has been studied by RNA-seq and marker-based sequencing (26). RNA-seq has been used to study the expression changes in the transcriptome before and after Poly (I:C) stimulation of rainbow trout embryo cells (26). Several differentially expressed genes (DEGs) have been found to be involved in multiple physiological systems, including endocrinal, reproductive, and immune systems. In a previous study, after the intraperitoneal injection of *P. shigelloides* solution, transcriptome sequencing was performed using the intestinal tissue samples of sturgeon. The results revealed that DEGs in the intestinal tissues were significantly enriched in biological processes, including the immune system process, immune effect process, and stimulus response (13). In the present study, a bacterium referred to as Cc2021 was isolated and purified as the key pathogenic bacterium from infected *C. carpio*. The pathogenic bacterium causing septicemia was identified through morphological observation, a regression challenge, biochemical identification, and 16S rRNA and gyrB gene sequence analysis. The mortality rate and effects of Cc2021 in *C. carpio* were analyzed on the basis of the tissue load, tissue edema, and histopathological findings. Comparative transcriptome analysis was performed to assess the immune response mechanism following Cc2021 challenge in both normal intestinal tissues and challenged intestinal tissues. The present study provides reference data regarding the pathogenic mechanism of *P. shigelloides* in *C. carpio*.

## Materials and Methods

### Ethical Statement

All experiments were conducted in accordance with the guidelines and regulations outlined by the Management and Use of Laboratory Animals of Hubei Province and complied with China’s existing laws and regulations for biological research. The present study did not involve any endangered or protected species.

### Fish

The experimental fish *C. carpio* (mean weight, 50 – 100 g) was obtained from the Guanqiao Experimental Station (Wuhan City, Hubei Province, China) and acclimatized at 25 ± 1°C. All the fish were fed twice daily with commercial pellets for 2 weeks before the experiments.

### Isolation and Cultivation of Bacterial Pathogens From Challenged *C. carpio*

Naturally infected *C. carpio* was obtained from the temperature-controlled Fish Culture Center of the Institute of Hydrobiology, Chinese Academy of Sciences (Wuhan City, Hubei Province, China). The body surface of *C. carpio* was disinfected with 70% alcohol and placed in a biosafety container for dissection. A sterilized inoculum ring was inserted deep into the lesion of the fish, the needle was gently rotated for sampling, and streaked inoculation was performed on the surface of sterile brain heart infusion (BHI) broth, followed by incubation at a constant temperature of 28°C for 48 h. A single dominant colony in the plate was selected and inoculated on another BHI plate thrice, and the purified pathogenic strain was obtained after pure culture. Finally, one part of the purified strain was used for subsequent experiments and the other part was stored in 50% glycerol at −80°C for future use.

### Biochemical Analysis of Pathogenic Bacteria

Bacteria were analyzed using bacterial biochemical microidentification tubes and test papers (Hangzhou Microbial Reagent. Co., Ltd. Hangzhou, China). A single colony was selected, inoculated in a microbiological reaction tube, and cultured at 28°C for 48 h. Following this, the biochemical indicators were tested (**Table 1**). The bacterial strains were preliminarily identified according to Bergey’s Manual of Systematic Bacteriology and Common Bacterial System Identification Manual (27).

**Table 1:** Physiological and biochemical identification of isolated strains.

Identification item	Result	Identification item	Result
Adipic acid	–	Lysine decarboxylase	+
Arginine double hydrolysis	+	Malic acid	+
Catalase	+	Maltose	+
Citric acid	–	Methyl red test	+
D-mannitol	–	Myo-lnositol	+
D-mannose	–	Phenylacetic acid	–
D-Xylose	–	Phenylalanine aminotransferase	–
D-fructose	+	Salicin	–
Fucose	+	Sorbitol	–
Glucose	+	Tryptophan deaminase	–
Lactose	+	Urease	–
L-rhamnose	–		

+ means positive; - means negative.

### Phylogenetic Analysis of 16Sr RNA and gyrB Genes From Pathogenic Bacteria

The 16S rRNA of the pathogenic bacterial strain was amplified using the 16S rRNA primers 27F (AGTTTGATCMTGGCTCAG) and 1492R (GGTTACCTTGTTACGACTT). The PCR cycling conditions were as follows: initial denaturation at 95°C for 5 min; followed by 35 cycles at 95°C for 30 s, annealing at 58°C for 30 s, and extension at 72°C for 1 min; and a final extension at 72°C for 10 min. The two primers used for PCR amplification of the gyrB gene sequence were as follows: UP1 (forward): 5′-GAAGTCATCATGACCGTTCTGCAYGCNGGNGGNAARTTYGA-3′ and UP2r (reverse): 5′-AGCAGGGTACGGATGTGCGAGCCRTCNACRTCNGCRTCNGTCAT-3′. The PCR reaction conditions were as follows: pre-denaturation at 94°C for 5 min, denaturation at 94°C for 1 min, renaturation at 60°C for 1 min, and extension at 72°C for 1 min. After 30 cycles, incubation was performed at 72°C for 7 min. The reaction process was ended at 16°C, followed by preservation at 4°C. Amplification products were placed in 1% agarose gel for electrophoresis. Positive amplification products were recovered and sequenced by TSINGKE Biotechnology Co., Ltd (Wuhan, Hubei, China). The obtained 16S rRNA and gyrB gene sequences were analyzed by the NCBI BLAST retrieval system for sequence homology, and multiple alignments were made with the sequences of strains with high sequence similarity obtained from the GenBank database using ClustalX software. The neighbor-joining method was used to construct phylogenetic trees using Molecular Evolutionary Genetics Analysis (MEGA) 7.0 software. A bootstrap test was performed with 1000 repetitions.

### Mortality Rate and Regression Challenge

The pathogenic bacterial strain was inoculated in BHI broth and cultured at 28°C under 200 rpm for 48 h. To determine the 50% lethal concentration of the pathogenic bacterial strain, four groups of *C. carpio* (n = 30) were intraperitoneally injected 100 μL of the bacterial strain (diluted in physiological saline) at concentrations of 1.0 × 10^7^ CFU/mL, 5.0 × 10^7^ CFU/mL, 1.0 × 10^8^ CFU/mL, and 5.0 ×10^8^ CFU/mL, while the control group was injected with sterile physiological saline (0.65% NaCl). Following this, mortality was monitored for the next 7 days.

After infecting the experimental fish (n = 100) with the measured half lethal concentration, the surviving fish were sampled from 0 to 7 days after the challenge. The blood, liver, head kidney, spleen, trunk kidney, and intestine (hindgut) of the challenged fish were randomly collected for the subsequent experiment (n = 5). Fish tissues injected with the same dose of saline were used as controls.

### Tissue Bacterial Load and Edema Detection

In total, 0.5 g of ground viscera (liver, head kidney, spleen, and intestine) was weighed and homogenized in BHI medium under sterile conditions using a Dounce tissue grinder (Sigma, USA). Serial dilutions (10^−4^, 10^−5^, and 10^−6^) were performed, 100 μL of the diluted sample was plated on BHI plates and incubated at 28°C for 48 h. Finally, the colony morphology was identified when the colonies were counted. CFU/g (log10) was calculated taking the dilution and total weight of the fish into consideration.

The whole target tissue was gathered and rinsed, and the immediate weight was recorded as the wet weight. Subsequently, the tissues were air dried for 2 days at 60°C, and their weight was recorded as the dry weight. The wet/dry weight ratio for each fish was calculated for evaluating tissue edema.

### Histological Analysis by Hematoxylin and Eosin Staining

Tissue samples from the head kidney, spleen, liver, and intestine were dissected and immediately fixed in 10% neutral buffered formalin at 4°C, dehydrated in a graded ethanol series, leaned in xylene, embedded in paraffin, and cut into 4 μm sections using a rotary microtome (Leica, Germany). The sections were stained with HE staining for routine histological examination and observed using a light microscope (Olympus BX51, Japan).

### Serum Biochemical Indices

Experimentally infected *C. carpio* was anesthetized with 3-aminobenzoic acid ethyl ester methanesulfonate (MS-222). Blood samples were collected from the caudal vein and placed for 1 h at room temperature. After centrifugation under 4500 rpm at 4°C for 15 min, the serum was collected and stored at −80°C. The serum biochemical indices of complement C3, superoxide dismutase (SOD), and lysozyme (LZM) were assessed using corresponding commercial kits (Nanjing Jiancheng Bioengineering Institute, Nanjing, China). The detection method refers to the kit instructions and previous reports (28).

### RNA Isolation, cDNA Library Preparation, and Sequencing

Total RNA was extracted from six tissue samples (three from the control groups and three from the challenge groups, collected from the intestinal tissue after 3 days of *P. shigelloides* challenge) using TRIzol Reagent (Invitrogen, USA) and then treated with RNase-free DNase I (Thermo Scientific, USA) at 30°C for 30 min to remove genomic DNA contaminants. Subsequently, the purity, quantity, and integrity of the extracted total RNA samples were assessed using a NanoDropND-2000 spectrophotometer (Thermo, Waltham, USA), by 1.2% (w/v) agarose gel electrophoresis, and using an Agilent 2100 Bioanalyzer (Agilent Technologies, Richardson, USA), respectively. RNA samples with an RNA integrity number (RIN) > 8, 28S/18S > 0.7, and A260/280 of approximately 2.0 were used to construct the RNA-seq library. Poly (A) mRNA was isolated from total RNA using poly (dT) oligo-attached magnetic beads, and cDNA libraries were prepared using the TruSeq RNA Sample Preparation Kit (Illumina, USA). In total, six cDNA libraries were sequenced using the Illumina Nova-Seq (BGI) sequencing platform to generate 150-bp paired-end reads.

### Transcriptome Quality Control and Gene Annotation

Read quality of the collected RNA-seq data was assessed using FastQC (http://www.bioinformatics.babraham.ac.uk/projects/fastqc/) (29). The filtered clean data were subsequently mapped to the *C. carpio* genome (NCBI: ASM1834038v1) using HISAT2 v2.1.0 (30). All genes were annotated in the genome sequences available in six public databases, including NCBI’s non-redundant protein sequence (Nr) database, Swiss-Prot, Kyoto Encyclopedia of Genes and Genomes (KEGG) (31), Gene Ontology (GO) (32), evolutionary genealogy of genes: Non-supervised Orthologous Groups (eggNOG) (33), and Pfam (34).

### Differential Gene Expression Analysis and Enrichment Analysis

DEGs between the control and challenge groups were identified using Baggerly’s test to calculate the fragments per kilobase of exon per million fragments mapped (FPKM) values. Benjamini–Hochberg correction was used to adjust the original *P*-values in Baggerly’s test in order to minimize the false discovery rate (FDR) (35). DEGs were identified if the associated PFDR was less than 0.05, and an absolute value of log2 (fold change) > 1 was regarded as the cut-off criterion. Clustering analysis was performed using the pheatmap package in R based on the FPKM values of DEGs. Subsequently, DEGs were enriched and subjected to GO term (36) and KEGG pathway analyses using clusterProfiler (37). A *P*-value < 0.05 indicated statistical significance, and the top 10 terms related to immunity were selected for visualization.

### qRT-PCR Validation of DEG Expression

In total, six genes associated with immune-related gene testis development were randomly selected for qRT-PCR to validate the RNA-seq results. Reversed-transcribed cDNA obtained from total RNA used for transcriptome sequencing was synthesized using the PrimeScript™ RT Reagent Kit with gDNA Eraser (Takara, Shanghai, China), according to the manufacturer’s protocol. All cDNA samples were diluted to 5 ng/µL and stored at −80°C until use. Specific primers were designed on the basis of NCBI Primer-BLAST (NCBI, USA), as listed in **Table S1**. All qRT-PCR reactions were performed in triplicate, and target specificity was determined on the basis of dissociation curve analysis. β-actin was selected as the internal control to normalize the expression level of each gene. The relative expression level of the target gene versus the β-actin gene was calculated using the 2^−ΔΔCT^ method. The obtained data were statistically analyzed using GraphPad Prism 7.0 software.

### Statistical Analysis

The results have been reported as the means ± SEs after data preparation and statistical analysis using GraphPad Prism 7.0 software. Statistical significance of the findings in each experimental group relative to the control group was assessed using Student’s two-tailed t-test. Significance (*P*-value) has been indicated as follows: *(*P* < 0.05), **(*P* < 0.01), and ***(*P* < 0.001).

## Results

### Isolation and Identification of Pathogenic Bacteria From Infected *C. carpio*

**Figure 1:**
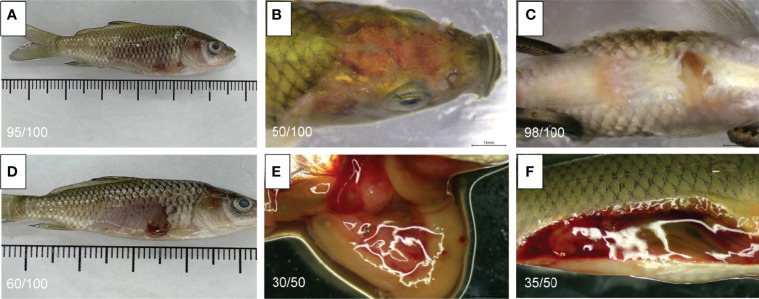
Symptoms of infected *C carpio*. **(A)** The abdominal scales of infected *C carpio* fell off, and the abdomen was red and swollen. **(B)** Red head of infected *C carpio*. **(C)** Swollen abdomen of infected *C carpio*. **(D)** The abdominal scales of infected *C carpio* fell off. The abdomen became red, with abscess-like fluid flowing out of it. **(E)** The visceral tissue was diffuse, and the liver was enlarged and pale. **(F)** The abdominal cavity of *C carpio* had a lot of yellowish fluid after dissection.

The symptoms of infected fish are shown in **Figure 1**; the symptoms mainly manifested as a mild floating head, outgroup swimming, slow swimming, and reduced feeding. In the middle stage of the disease, the fish stopped feeding completely, with slight eyeball protrusion, slight bleeding in the jaw and abdomen, and redness of the head (**Figures 1A, B**). In the later stage, death, abdominal bleeding, and swelling were noted (**Figures 1C, D**). After dissection, it was found that the dead fish had fluid accumulation (ascites) in the abdominal cavity. Moreover, the liver was swollen, with light yellow and irregular red spots distributed on it. Furthermore, the tissue felt slightly brittle, like bean paste (**Figures 1E, F**).

**Figure 2:**
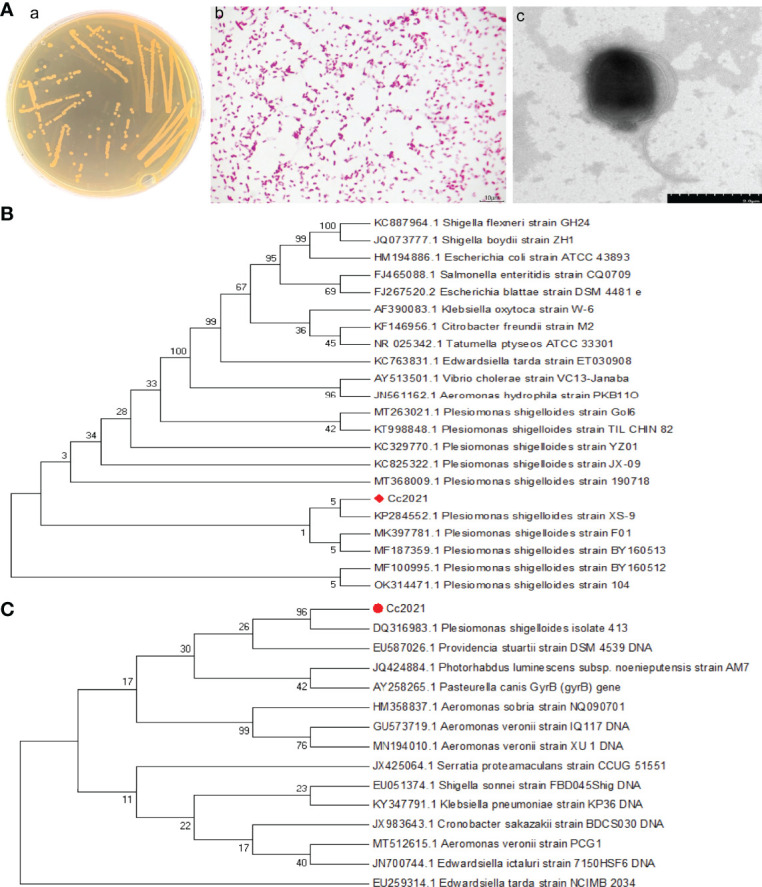
Identification of the pathogen’s morphological and molecular characteristics. **(A) (a)** Growth characteristics of isolates in BHI medium. **(b)** Gram staining results of isolates. **(c)** Negative staining results of isolates. **(B)** Phylogenetic tree based on the partial 16S rRNA gene sequences included in the expanded MicroSeq 500 library; the algorithm used to construct the tree was the unweighted pair group method using averages (UPGMA). **(C)** Phylogenetic tree based on the partial gyrB gene sequences included in the expanded MicroSeq 500 library; the algorithm used to construct the tree was UPGMA.

The dominant strain of pathogenic bacteria was isolated from the lesions of *C. carpio* (ulceration, ascites, hepatopancreas, and intestinal tract) and named Cc2021 (27). The bacterium grew well on BHI agar, and the colonies were round, beige opaque, smooth, moist, and slightly raised but with neat edges (**Figure 2Aa**). Gram staining results were microscopically observed. The bacterial cells stained red and appeared as short rods, which were arranged singly or in pairs, indicating that the strain was gram negative (**Figure 2Ab**). Negative stain electron microscopy revealed a straight rod with a terminal round shape, no spore or capsule, and two fascicular flagella at both ends. The size of the bacterium (short diameter × long diameter) was 0.8–1.0 μm × 3.0 μm (**Figure 2Ac**). These characteristics were consistent with the description provided in Bergey’s Manual of Systematic Bacteriology.

The physiological and biochemical characteristics of the pathogen are provided in **Table 1**. According to Bergey’s Manual of Determinative Bacteriology (9th ed.), the bacterium could hydrolyze arginine, D-fructose, fucose, glucose, and myo-inositol but not adipic acid, citric acid, D-mannitol, phenylalanine aminotransferase, phenylacetic acid, sucrose, salicin, sorbitol, and urease. The results of methyl red and malic acid tests were also positive.

To further identify the strain, its 16S rRNA and gyrB genes were sequenced. NCBI BLAST was used for homology analysis based on the 16S rRNA and gyrB gene sequences of the strain; the results revealed that the strain was 99% similar to *P. shigelloides*. Phylogenetic analysis was performed after screening sequences with high similarity in terms of sequence alignment; the results (**Figures 2B, C**) revealed that both 16S rRNA and gyrB gene sequences of *P. shigelloides* formed a single cluster. Therefore, based on its morphological, physiological, biochemical, and molecular characteristics, the strain was identified to be *P. shigelloides*.

### Pathogenicity of *P. shigelloides *in *C. carpio*

**Figure 3:**
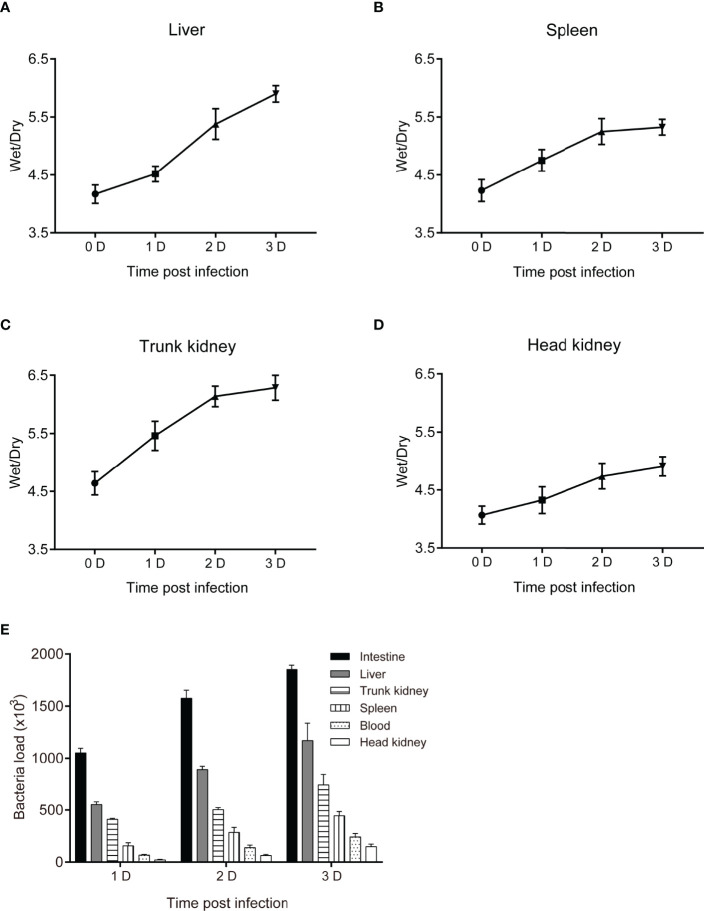
Tissue edema and bacterial load in *C. carpio* after *P. shigelloides* challenge. **(A–D)** The dry/wet ratio of the liver, spleen, body kidney, and head kidney of *C. carpio* 0–3 days after *P. shigelloides* challenge. **(E)** Bacterial load in *C. carpio* tissues after *P. shigelloides* challenge.

To assess the pathogenicity of *P. shigelloides* in *C. carpio*, one group of *C. carpio* was injected with physiological saline (65% NaCl) (control group), while four groups were injected with four different concentrations (1.0 × 10^7^ CFU/mL, 5.0 × 10^7^ CFU/mL, 1.0 × 10^8^ CFU/mL, and 5.0 × 10^8^ CFU/mL) of the pathogenic bacterial strain (experimental groups). Subsequently, deaths were recorded (**Table 2**). Both high and low concentrations of the bacterial suspension resulted in typical symptoms, similar to those observed in naturally infected fish. After the injection of a high concentration (5.0 × 10^8^ CFU/mL) of the bacterial suspension, a large number of fish died within 2 days (73.3%), with the mortality reaching 100% within 7 days. The mortality rates of *C. carpio* were 80%, 53.3%, and 23.3% after the injection of 1.0 × 10^8^ CFU/mL, 5.0 × 10^7^ CFU/mL, and 1.0 × 10^7^ CFU/mL concentrations, respectively. On the other hand, no death was noted in the control group. These mortality results served as the basis for the follow-up regression challenge experiment. Tissue edema and bacterial loads in *C. carpio* were assessed after *P. shigelloides* challenge. The dry/wet weight ratios of the liver, spleen, head kidney, and body kidney of *C. carpio* showed an increasing trend with an increase in the challenge time. After 2 days post-challenge, the dry/wet weight ratios of the liver and head kidney gradually increased, while those of the spleen and head kidney tended to decrease (**Figures 3A–D**). The bacterial load in the tissues from the intestine, hepatopancreas, head kidney, body kidney, and spleen of *C. carpio* infected with *P. shigelloides* and the blood bacterial concentrations increased with an increase in the challenge time. The bacterial load in the intestinal tissues was the highest, followed by that in the tissues from the hepatopancreas, head kidney, and spleen. The lowest bacterial load was noted in the blood and body kidney tissues (**Figure 3E**).

**Table 2:** Death statistics of *C. carpio* infected with *P. shigelloides*.

	1 D	2 D	3 D	4 D	5 D	6 D	7 D
Physiological	0	0	0	0	0	0	0
1.0 × 10^7^	2	3	5	5	6	7	7
5.0 × 10^7^	4	6	9	9	13	15	16
1.0 × 10^8^	9	11	17	18	20	23	24
5.0 × 10^8^	13	22	28	29	30	30	30

### Histopathological Changes in *C. carpio* After *P. shigelloides* Challenge

**Figure 4:**
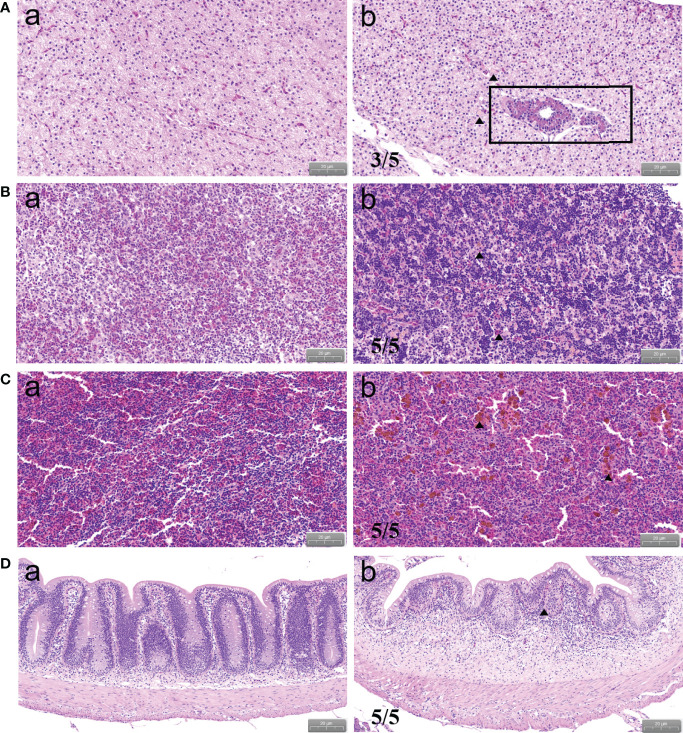
Histopathological changes in *C carpio* tissues after *P. shigelloides* challenge. **(A)** Infected liver. **(B)** Infected head kidney. **(C)** Infected spleen. **(D)** Infected intestine. Pictures of typical symptoms are shown. Scale bar of A, B, and C = 20 μm. Scale bar of D = 50 μm.

To observe the histopathological state of *P. shigelloides*-infected *C. carpio*, HE staining was performed using tissues from the liver, head kidney, spleen, and intestine at each period. The typical pathological states are shown in **Figure 4**. In the control group, the hepatic cells were rich in cytoplasm, had an obvious nucleus and cytoplasm, had a clear cell boundary, and were evenly dispersed **(Figure 4Aa**). After the artificial challenge, the inflammatory cells in the liver of infected *C. carpio* showed focal aggregation, with an unclear structure and a fuzzy boundary. The hepatic cells were denatured and necrotic, and the nucleus and cytoplasm were concentrated. Some hepatic cells were vacuolated and had dissolved nuclei. The tissue was loose and had edema, and degeneration and necrosis were noted around the sinuses. Moreover, the tissue structure was destroyed and several hepatocytes were vacuolated (**Figure 4Ab**). The renal structure of infected *C. carpio* was disorganized, with swelling and degeneration of cells, a narrow renal cavity, and a transparent tube type. The renal tissue was filled with lymphocytes, accompanied by multiple bleeding spots and an obvious inflammatory reaction (**Figure 4Bb**). Moreover, ferriflavin deposition in the splenic tissue, atrophy of the white pulp, and a reduced area occupied by splenic pulp hyperemia were noted (**Figure 4Cb**). The intestinal structure of *C. carpio* in the control group was intact; the inner wall of the tissue was not damaged, intestinal epithelial villi were arranged tightly and in an orderly manner, and basal epithelial tissues were obviously stratified (**Figure 4Da**). Villus epithelial cells in the intestinal tissues of infected *C. carpio* fell off, with the infiltration of local lymphocytes and the swelling of and increase in goblet cells in the mucosal epithelium being noted (**Figure 4Db**).

### Changes in Serum Biochemical Indices of *C. carpio* After *P. shigelloides* Challenge

**Figure 5:**
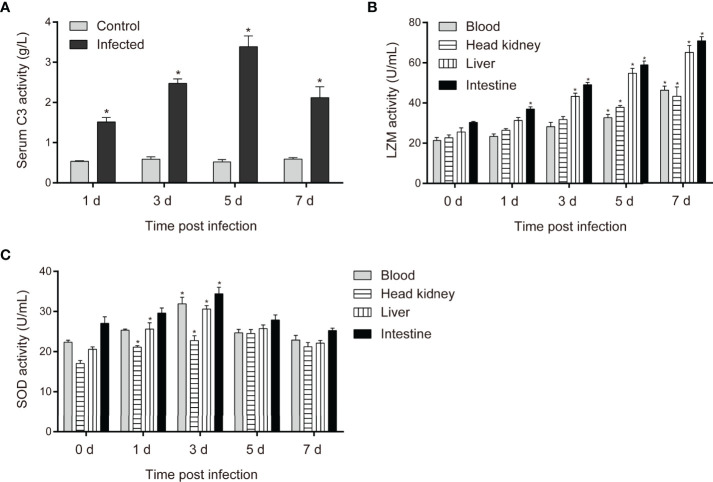
**(A)** Changes in complement C3 levels in the serum of *C carpio* with time after *P. shigelloides* challenge. **(B)** Changes in LZM activity in tissues of *C carpio* with time after *P. shigelloides* challenge. **(C)** Changes in SOD activity in tissues of *C carpio* with time after *P. shigelloides* challenge. "*" means P < 0.05.

To assess the degree of activation of antibacterial immune responses in the key immune organs and bloodstream of *C. carpio* during *P. shigelloides* challenge, C3, SOD, and LZM activities were assessed at different stages after the challenge. The expression level of C3 in the serum increased from 1 to 7 days and reached its maximum on the 5th day post-challenge, becoming nearly three times the normal expression level (**Figure 5A**). The expression levels of LZM in all the tissues of healthy common carp were similar. The LZM levels in the blood, head kidney, liver, and intestine significantly increased after the challenge. Moreover, the LZM levels in all the tissues continued to increase from 1 to 7 days post-challenge. LZM activity was the highest in the intestine, followed by the liver and blood, while it was the lowest in the head kidney after the challenge (**Figure 5B**). The SOD levels in each tissue of *C. carpio* showed a trend of first increasing and then decreasing after the challenge; the increase was most obvious on 1 and 3 days, followed by a decrease (**Figure 5C**). The highest SOD activity was noted in the intestinal tissue after the challenge; this may be related to enteritis caused by the bacterium.

### Transcriptome Sequencing and Annotation

All raw data generated in the present study have been uploaded to the NCBI Sequence Read Archive (SRA) under the registration number PRJNA793911. The original sequencing data included reads having low quality, joint contamination, and a high unknown base N content, which needed to be removed before data analysis in order to ensure the reliability of the results. After sequence filtration, a comparison of all genomes was performed, as shown in **Figure S1A**. Expression profiles of all the genes were obtained in both the control and challenge groups, with half reaching at least one FPKM (**Figure S1B**). Correlation analysis of the samples revealed that the correlation coefficients of Challenge 2 and Control 1 within the group were far less than 0.7; thus, these two samples were removed from subsequent analysis (**Figure S1C**). Analysis of FPKM expression levels for all the genes in the visualized heatmap revealed that the expression levels of Control 2 and Control 3 genes were consistent and those of Challenge 1 and Challenge 3 genes were consistent (**Figure S1D**).

To further assess the immune response mechanism of *C. carpio* during *P. shigelloides* challenge, comparative transcriptome analysis was performed for comparing the gene expression profiles in the intestinal tract of *C. carpio* between the control and challenge groups. For this analysis, three control group samples and three challenge group samples, collected on the 3rd day after the infection, were included. Based on sequence homology, GO enrichment analysis of DEGs in the intestinal tract was performed after *P. shigelloides* challenge, and the similarities and differences in these terms were analyzed. To further understand the functions of DEGs and the signaling pathways that they participate in, all DEGs were classified into three GO categories: biological process (BP; 20 subclasses), molecular function (MF; 20 subclasses), and cellular component (CC; 20 subclasses) (**Figure S2A**). KEGG pathway analysis of the intestinal tissue after *P. shigelloides* challenge revealed that there were 21 KEGG metabolic pathways involving different genes, mainly including the complement and coagulation cascade pathway, TNF signaling pathway, interleukin (IL)-17 signaling pathway, and toxoplasmosis and systemic lupus erythematosus pathway (**Figure S2B**).

### Identification of Candidate Genes Responding to *P. shigelloides* Infection

**Figure 6:**
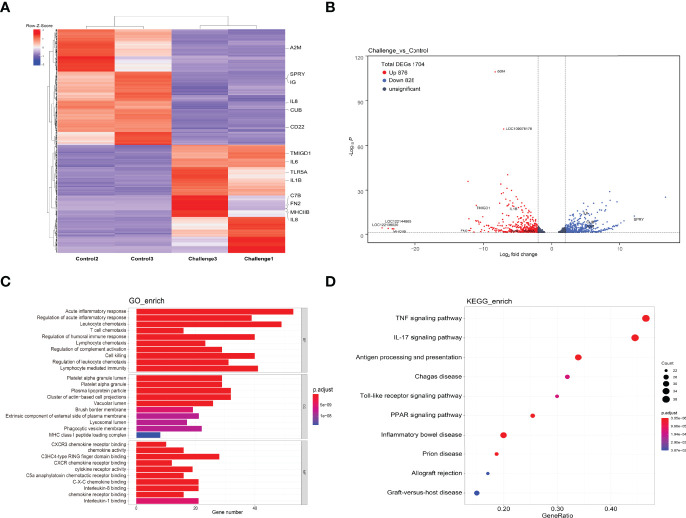
Differentially expressed genes. **(A)** Heatmap showing the expression of differentially expressed genes in each sample. **(B)** Volcano map showing the expression of differentially expressed genes in each group of samples. **(C)** GO enrichment analysis was performed for differentially expressed genes, and three categories of the top 10 immune-related pathways were selected for display. **(D)** KEGG enrichment analysis was performed for differentially expressed genes, and the top 10 immune-related pathways were selected for display.

The identification of DEGs can provide a deeper understanding of the changes in the immune mechanism of *P. shigelloides-*infected *C. carpio*. The heatmap in **Figure 6A** shows the expression of DEGs in each group. Intestinal gene expression was higher in the challenge group. Importantly, there was no significant difference in DEGs between the control and challenge groups. In total, 1704 genes were differentially expressed in the control and challenge groups, including 876 upregulated genes and 828 downregulated genes (**Figure 6B**). The upregulation level of genes was thus significantly higher than the downregulation level, which may be attributed to the cascade amplification of immune signal transmission.

To further assess the immune response after *P. shigelloides* challenge, we focused on immune-related DEGs classified into BP, MF, and CC categories, as shown in **Figure 6C**. The top 10 KEGG pathway terms are shown in **Figure 6D**. GO enrichment analysis of this module was dominated by functional categories related to immune system. In the BP category, acute inflammatory response, regulation of acute inflammatory response, and leukocyte chemotaxis were the top three terms. In the CC category, platelet alpha granule lumen, platelet alpha granule, and plasma lipoprotein particle were the top three GO terms. In the MF category, CXCR3 chemokine receptor binding, chemokine activity, and C3HC4-type RING finger domain binding were the top three terms. Furthermore, KEGG pathway enrichment of immune-related DEGs was analyzed. The immune-related signaling pathways of all enriched immune-related DEGs were selected as the top 10 ones, e.g., the TNF signaling pathway, IL-17 signaling pathway, antigen processing and presentation pathway, Chagas disease pathway, and Toll-like receptor (TLR) signaling pathway (**Figure 6D**). Some immune-related DEGs are shown in **Table 3**. These results suggested that both the innate and adaptive immune responses of *C. carpio* played vital roles in its response to *P. shigelloides* challenge. The discovery of the immune-related pathways and genes could provide a theoretical basis to understand the molecular mechanism of *C. carpio* infected with *P. shigelloides* or other bacteria.

**Table 3:** Immune-related differentially expressed following *P. shigelloides* challenge based on the KEGG analysis.

Category/gene ID	Gene name	log2 (Fold changes)	P-value	Description
**TNF signaling pathway**				
109062992	IL1b	+5.730787135	3.14E-23	interleukin 1, beta
109076179	IL-8	-5.535978561	2.67E-11	C-X-C motif chemokine 11-6
109053615	ifng1	-3.790176668	0.00181126	interferon gamma 1
109102164	fosl1a	4.159704917	0.0000436	FOS like 1, AP-1 transcription factor subunit a
109080931	bZIP_1	-3.906674161	0.000232279	V-fos FBJ murine osteosarcoma viral oncogene homolog
**IL-17 signaling pathway**				
109070669	c8g	- 3.812342307	0.000618488	complement component 8, gamma polypeptide
109095592	bZIP_2	+ 3.367885457	4.31E-20	CCAAT/enhancer-binding protein delta-like
109071632	ptgs2a	+2.9484497	8.02E-05	prostaglandin-endoperoxide synthase 2a
109076910	Hsp90	+2.838544283	0.000653388	heat shock protein HSP 90-alpha 1-like
109090204	ptgs1	+2.80024523	7.04E-06	prostaglandin-endoperoxide synthase 1
**Antigen_processing_and_presentation**				
109057161	hsp70.3	+7.645087149	3.74E-05	heat shock cognate 70-kd protein, tandem duplicate 3
109084538	hsp70	+6.7174199	0.000418312	heat shock 70 kDa protein
109053615	IFN-gamma	-3.610905272	0.001395776	interferon gamma 1
109112543	MHC_II_beta	-3.488986733	0.000170372	beta-2-microglobulin-like
122149025	HSP90	+2.128154638	0.001997698	heat shock protein HSP 90-alpha-like
**Chagas_disease**				
109047863	alpha	+6.636005348	3.94E-09	guanine nucleotide-binding protein G(o) subunit alpha-like
109074247	A2M	-5.348810882	9.51E-06	complement C3-like
109092994	serpine1	+2.585673392	3.95E-05	serpin peptidase inhibitor, clade E (nexin, plasminogen activator inhibitor type 1), member 1
109069312	c1qc	-2.281829004	8.04E-08	complement component 1, q subcomponent, C chain
109079578	G-alpha	+2.478708135	1.58E-09	guanine nucleotide-binding protein G(olf) subunit alpha-like
**Toll_like_receptor_signaling_pathway**				
109044888	tlr5a	+3.452532589	4.41E-06	toll-like receptor 5a
109113360	jun	+1.843941536	0.000150488	Jun proto-oncogene, AP-1 transcription factor subunit
109053776	IL8	-1.706187902	0.000284318	C-C motif chemokine 3-like
109045134	C2-set_2	+1.354523626	0.002162804	T-lymphocyte activation antigen CD80-like
109078735	IRF	-1.343814554	0.001013223	interferon regulatory factor 8-like

+ means up; - means down.

### Verification of Immune Response Candidates Activated by *P. shigelloides*

**Figure 7:**
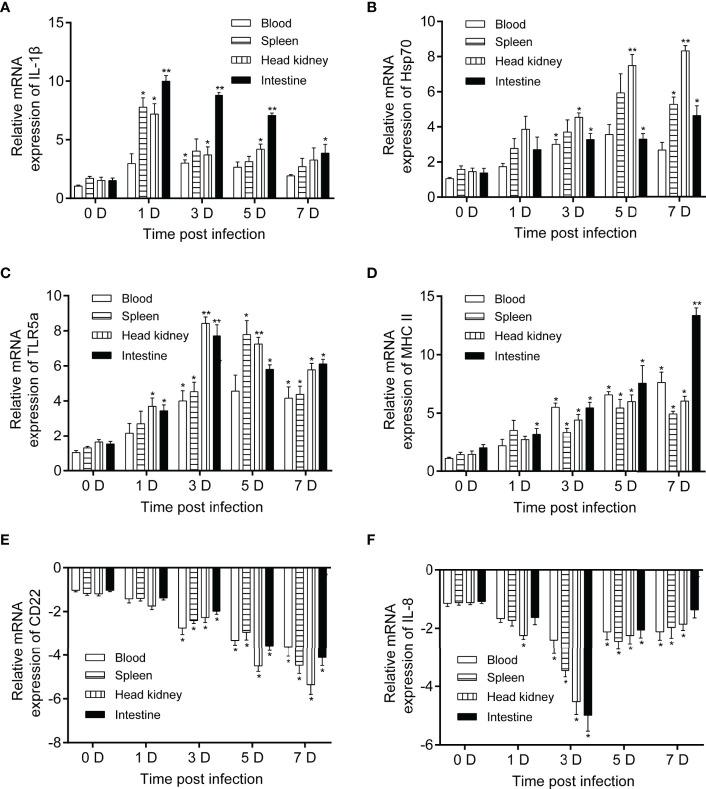
Relative mRNA expression levels of IL-1β, Hsp70, TLR5a, MCH II, CD22, and IL-8 in *C carpio* tissues after *P. shigelloides* challenge. mRNA expression levels of IL-1β **(A)**, Hsp70 **(B)**, TLR5a **(C)**, MCH II **(D)**, CD22 **(E)**, and IL-8 **(F)** in the blood, spleen, head kidney, and intestine of *C carpio* at 0, 1, 3, 5, and 7 days after *P. shigelloides* challenge. The β-actin gene was used as a reference gene, and the data of day 0 were used for comparison with those of days 1, 3, 5, and 7 in order to analyze the differences. Data are presented as the means ± SEs (n = 4). Statistical analysis was performed using unpaired Student’s t-test (*P < 0.05 and **P < 0.01).

To verify significant immune response candidates activated by *P. shigelloides*, qRT-PCR was performed on several candidates selected from the comparative transcriptome. The expression levels of IL-1β, Hsp70, TLR5a, MCH II, CD22, and IL-8 were verified in the blood, spleen, head kidney, and intestine at 0, 1, 3, 5, and 7 days after the challenge (**Figure 7**). The mRNA expression level of IL-1β dramatically increased and immediately reached the highest level on the 1st day after the challenge, following which it began to decrease gradually. The expression level of IL-1β was the highest in the intestine (**Figure 7A**). The mRNA expression level of Hsp70 gradually increased in all immune tissues from 1 to 7 days after the challenge; the expression level was higher in the head kidney and spleen (**Figure 7B**). The mRNA expression level of TLR5a was upregulated to a similar extent in all the tissues after the challenge, with the increase being the highest at 3 and 5 days (**Figure 7C**). Moreover, the mRNA expression level of MHC II was continuously upregulated after the challenge, with the most significant increase being noted in the intestine (**Figure 7D**). In contrast, the mRNA expression levels of CD22 and IL-8 were downregulated to varying degrees after the challenge in all the tissues (**Figures 7E, F**).

## Discussion

*P. shigelloides*, belonging to the order Eerobacterales, is widely distributed in nature. it is a kind of global distribution bacteria, its detection rate has obvious seasonal, high detection rate in summer, which may be related to the optimal growth temperature of the bacteria is higher. And *P. shigelloides* is a new pathogen of digestive tract inflammation in many aquatic animals, as well as a zoonotic pathogen, which is a pathogen of gastrointestinal infection outbreak transmitted by water and food (7). In recent years, there have been increasing reports on the pathogenicity of *P. shigelloides* in fishes. The pathogenic symptoms of *P. shigelloides* are slightly different in different fishes; this may be caused by the differences in fish species, culture environments, and strains (38, 39). However, few studies have been conducted on the pathogenic mechanism of *P. shigelloides* in fishes (40). Studies on the isolation and identification of bacterial species are essential for understanding the exact incidence of a disease in a given region and for planning epidemiological studies and control and eradication programs (41). Moreover, understanding how bacteria cause pathological symptoms can form the basis of preventing and treating bacterial diseases (42, 43). In the present study, we isolated a bacterium from *C. carpio* and identified it to be *P. shigelloides* by morphological, physiological, and biochemical methods; gene sequence analysis; and PCR. In addition, we used the isolated *P. shigelloides* strain to infect *C. carpio* and studied the changes in histopathology, tissue bacterial load, tissue edema, and immune gene expression to understand its pathogenic mechanism. Finally, and most importantly, through high-throughput sequencing technology, we explored the pathogenic mechanism of *P. shigelloides* and the immune response mechanism of *C. carpio* after *P. shigelloides* challenge. Our findings can serve as a reference for drug designing and vaccine adjuvant screening against pathogens.

*P. shigelloides* is widely distributed and found in different water bodies, animals, and humans (7). It can cause human acute gastroenteritis and other toxic symptoms. In recent years, it has been detected in different aquatic animals and has been found to be highly pathogenic to fishes. It can multiply in the intestines of fishes, damaging their organs and ultimately causing death (37). Grass carp mainly presents with redness and swelling of the body surface, liver, intestine, and anus (38, 39). *Garra rufa* infected with *Streptococcus agalactiae* presents with surface ulceration and intestinal and hepatic lesions (40). Pathological changes in the liver, stomach, intestine, kidney, throat, and other organs have been reported to be caused in *Trionyx sinensis* by *Aeromonas hydrophila*, with the findings in male and female gonads also being inconsistent (39). However, acute diarrhea and food poisoning have been found to be the main symptoms in humans after *P. shigelloides* challenge (41). In the present study, the disease in *C. carpio* was characterized by an abdominal cavity, the emergence of a few different levels of fish ascites, hepatomegaly, yellowish color, and an irregular distribution of red dots. Anatomic assessment revealed a crispy organization, light inflammation of the gills, erosion and rotting of the gills, bleeding of the gills, and significant intestinal inflammation. *P. shigelloides* is a pathogen that causes zoonotic diseases. It also causes a wide range of clinical symptoms in humans, such as acute gastroenteritis and diarrhea (7). Therefore, it is an important pathogen that can pose a risk to public health, especially to people who directly touch any infected fish.

Complement C3, SOD, and LZM in the serum are powerful indicators of the non-specific immune response of aquatic animals, which can identify pathogens, inactivate viruses, and reflect the degree of somatic cell damage to a certain extent (18). Moreover, they play an important role in the non-specific immunity of fishes (44). The complement system is an important component involved in the resistance of fishes to challenge and is involved in target cell lysis and conditioning reactions after activation. Complement C3 is the main component of the complement system (45). A previous study reported an increase in the complement C3 level of hybrid tilapia fed different doses of the commercial product DVAQUA (46). The serum complement C3 level of yellow catfish was found to significantly increase at 7, 14, 21, and 28 days after the injection of inactivated *E. ictaluri* vaccine, further providing that the vaccine could improve the non-specific immunity of yellow catfish (47). However, in the present study, the complement C3 level in the serum of *P. shigelloides*-infected *C. carpio* significantly increased, reached its maximum after 5 days post-challenge, and then significantly decreased. The results revealed that the non-specific immune mechanism was activated in *C. carpio* after *P. shigelloides* challenge and was maintained for a long time. SOD activity has been found to be closely related to the immune level of an organism. The SOD activity of *Channa argus* increased from 9 days after the injection of inactivated *Nacardia seriolea*, reaching its peak on the 12^th^ day (48). In the present study, the SOD activity in the serum of *C. carpio* first increased after the injection of *P. shigelloides* and then decreased after reaching its maximum on day 3. This result indicated that *C. carpio* could produce a large number of free radicals, such as superoxide anions, at an early stage of the challenge and improve its antioxidant and immune capacity to resist the challenge. Moreover, LZM activity was found to increase in *A. hydrophila*-infected Chinese sturgeon (49). This result was consistent with that of the present study. In conclusion, the isolated bacterial strain could stimulate the body to produce an immune response after a challenge, which was manifested as increased C3, SOD, and LZM activities in the serum of *C. carpio*; this in turn stimulated the non-specific immunity of *C. carpio* to a certain extent.

Intestinal mucosal immunity is an important mechanism by which a host can resist a pathogen; it plays an important role in natural immunity (50). In previous studies, *P. shigelloides* challenge was found to destroy the intestinal tissue structure, reduce the number of mucosal immune-related cells, cause intestinal digestion and absorption dysfunction, and activate the mucosal immune response in hybrid sturgeon (12, 17). In the present study, the intestinal transcriptome of *C. carpio* was altered by *P. shigelloides* challenge. The results revealed that there were 1704 DEGs in the intestinal tissues of *C. carpio* after *P. shigelloides* challenge, including 876 upregulated DEGs and 828 downregulated DEGs. This may be related to extensive bacterial replication in *C. carpio* and the resistance of *C. carpio* to bacterial challenge. These results indicate that bacterial invasion into the digestive mucosa of *C. carpio* can induce a specific immune response through a non-specific immune function of the body, i.e., the phagocytosis of immune cells. Some pathogens, such as *Legionella pneumophila*, *Listeria monocytogenes*, and *S.* Typhi, recognize cytoplasmic pathogen-associated molecular patterns (PAMPs). The RIG-1-like receptor signaling pathway is activated to promote the expression of type 1 interferons and pro-inflammatory cytokines, thereby protecting against bacterial challenge (51, 52). Fish TLRs (such as TLR3) are responsible for recognizing not only nucleic acids produced by viruses but also bacterial components, thereby acting as antibacterial agents (53). Intracellular parasitic bacteria, such as *L. monocytogenes* and *Mycobacterium tuberculosis*, can activate the cellular solute DNA sensing pathway by recognizing and activating the cGAS-STING pathway. Following this, the TBK1/IRF3 signal cascade is activated to activate the type I IFN transcriptional response and exert antibacterial effects (54). In the present study, the main immune-related signaling pathways in the intestine of *P. shigelloides*-infected *C. carpio* were the TNF signaling pathway, IL-17 signaling pathway, antigen processing and presentation pathway, Chagas disease pathway, and TLR signaling pathway. These signaling pathways play an important role in immune cell activation, inflammatory response, DNA damage, lipid metabolism, and the inhibition of pathogenic bacteria.

To further understand the antibacterial mechanisms triggered by the innate immune response in *C. carpio* after *P. shigelloides* challenge, we analyzed the expression levels of six DEGs related to immunity in different tissues and at different times. The IL family is one of the largest cytokine families that plays an important role in the innate and acquired immune responses of the host (55). IL-1β was the first IL to be identified. It plays a critical role in the initiation and regulation of immune and inflammatory responses in many economically important teleost fishes (55). IL-1β in teleost fishes also plays an important role in host resistance to pathogens (56). Recent research has revealed that IL-1β plays an important role in the immune response of large yellow crocea against *Vibrio alginolyticus* (55). In the present study, after the challenge, the mRNA level of IL-1β rapidly increased and reached the highest level on day 1, following which it began to gradually decrease. The expression level in the intestine was the highest. IL-8, a CXC-type chemokine, plays a key role in acute inflammation by recruiting neutrophils in mammals. Moreover, fish IL-8 is a functional homolog of mammalian IL-8 (57). IL-8 is a well-known cytokine that plays a crucial role in inflammatory responses and is activated in response to various diseases (57). IL-8 was found to be expressed in various tissues of unchallenged olive flounder. In particular, its expression was the highest in the gills (58). The same results have been reported in rainbow trout, tongue sole (*Cynoglossus semilaevis*), and common carp (59). In the present study, the mRNA expression levels of IL-8 were downregulated to varying degrees after the challenge in all the tissues. These results suggest that IL-8 is closely related to inflammation and immune regulation in olive flounders and may be used as a basis for research on the immune systems of other fishes. HSP70 is an important member of the heat-shock protein superfamily; it appears in almost all species. HSP70 proteins also play a role in improving disease resistance (60). In the present study, the mRNA expression level of Hsp70 gradually increased in all the immune tissues 1–7 days after the challenge. This upregulation was most obvious in the head kidney and spleen. These results suggest that HSP70 is involved in the immune response against bacterial attacks and heat stress. TLR5 is associated with flagellin detection (61). Moreover, significant upregulation of TLR pathway genes has been reported after *A. hydrophila* challenge in other fish species (61). Increased expression levels of tlr5a and tlr5b genes were noted in turbot (*Scophthalmus maximus* L.) mucosal tissues (i.e., intestine and gills) in response to infection by the gram-positive, non-flagellated pathogen *S. iniae* (62). In the present study, the mRNA expression level of TLR5a was upregulated to a similar extent in all the tissues after the challenge, with the increase being the highest at 3 and 5 days. A similar upregulation of tlr5 in response to alive and formalin-killed *R. salmoninarum* has been reported (63, 64). Therefore, the role of TLR5 beyond the recognition of flagellin, particularly after exposure to non-flagellated bacteria in teleosts, warrants further investigation.

In conclusion, in the present study, a comprehensive and accurate method was used to identify pathogenic bacteria in *C. carpio.* Changes in the tissue bacterial load, tissue edema, histopathological parameters, innate immunity, and survival rates of *P. shigelloides*-infected *C. carpio* during the septicemia process were systemically analyzed. The results indicated that the host response to *P. shigelloides* challenge involved an inflammatory reaction. To further assess the pathogenesis, comparative transcriptome analysis was performed between the challenge and control groups at 3 days post-challenge. The analysis revealed 1704 genes that were differentially expressed. Further analysis revealed that about 10 KEGG pathways were involved in the host immune response to bacterial challenge; these included the TNF signaling pathway, IL-17 signaling pathway, antigen processing and presentation pathway, Chagas disease pathway, and TLR signaling pathway, among others. The present findings can serve as a reference for understanding the pathogenic mechanism of *P. shigelloides* in *C. carpio* and broaden the understanding of the systemic immune response of *C. carpio*.

## Data Availability Statement

The datasets presented in this study can be found in online repositories. The names of the repository/repositories and accession number(s) can be found below: https://www.ncbi.nlm.nih.gov/, PRJNA793911.

## Ethics Statement

The animal study was reviewed and approved by Institute of Hydrobiology, Chinese Academy of Sciences. Written informed consent was obtained from the owners for the participation of their animals in this study.

## Author Contributions

DL conceived the project. DL and HC designed the study. HC and YZ performed most of the experiment and analyses. KC, YW, HL, and FL helped in experiment, data curation and analyses. YL helped in breeding and sampling. HC, YZ, DL, WH and ZZ prepared the draft and final version of the manuscript. All authors read and approved the final manuscript.

## Acknowledgments

This work was supported by grants from the National Natural Science Foundation of China (Grant No. 31922085 to DL), the Strategic Priority Research Program of CAS (Grant No. XDA24010108to DL), Natural Science Foundation of of Hubei Province (Grant No. 2020CFA056 to DL), China Postdoctoral Science Foundation on the 70th finance (Grant No. 2021M703436 to YZ), and Postdoctoral innovation research positions of Hubei Province (YZ). The authors would like to thank Rui Li, Hairong Liu, Kaifeng Meng, Wentao Zhu and Chuang Xu for technical advice and assistance in experiments, Yuan Xiao and Zhenfei Xing at the Analysis and Testing Center of Institute of Hydrobiology for their assistance with providing Transmission Electron Microscope analysis.

## Conflict of Interest

The authors declare that the research was conducted in the absence of any commercial or financial relationships that could be construed as a potential conflict of interest.

## Publisher’s Note

All claims expressed in this article are solely those of the authors and do not necessarily represent those of their affiliated organizations, or those of the publisher, the editors and the reviewers. Any product that may be evaluated in this article, or claim that may be made by its manufacturer, is not guaranteed or endorsed by the publisher.
