# Supplementary material for: Isolation, Identification, and Investigation of Pathogenic Bacteria From Common Carp (Cyprinus carpio) Naturally Infected With Plesiomonas shigelloides
Source: Front Immunol. 2022 Jun 30;13:872896. doi: 10.3389/fimmu.2022.872896 (PMC9279890; doi:10.3389/fimmu.2022.872896)
Supplement: Supplementary file 1 [file DataSheet_1.docx]

# Figure captions

Fig. S1. Overview and analysis of transcriptome samples. A. Comparison of genomes of all the samples. B. All the samples were based on the expression distribution of FPKM. C. Correlation analysis of all the samples. D. Heatmap showing the expression levels of FPKM in all the samples.

Fig. S2. A. GO enrichment analysis was performed for differentially expressed genes. B. KEGG enrichment analysis was performed for differentially expressed genes.

# Tables

## **Table S1. Primer sequences in this study.**

| Gene name | Primer direction | Primer sequence (5′-3′) | Size (bp) |
| --- | --- | --- | --- |
| CD22 | Forward | TAGGTGGCATACACCTCAGC | 116 |
|  | Reverse | TTCTGTGCCGCATGTCAATG |  |
| Hsp70 | Forward | AAACAGTCTGACCTGACGAAGA | 117 |
|  | Reverse | TGCCCAGGTCAATGCCAATA |  |
| IL-1b | Forward | CACCCGCTGGATTTGTCAGA | 114 |
|  | Reverse | GTCGCATTGGCAACTCATGG |  |
| MHCII | Forward | TGTCACTGCTACAGTACTTGAGTT | 80 |
|  | Reverse | GTACTGTTGAACACTTTTTGCTCC |  |
| TLR5a | Forward | CCTTGCCGCAAACCTCAAAA | 148 |
|  | Reverse | ATGTTCGTCATCGGGGCAAT |  |
| IL-8 | Forward | GAAGAATGGTGCAGGACGGA | 145 |
|  | Reverse | GAAGAATGGTGCAGGACGGA |  |

# Figures


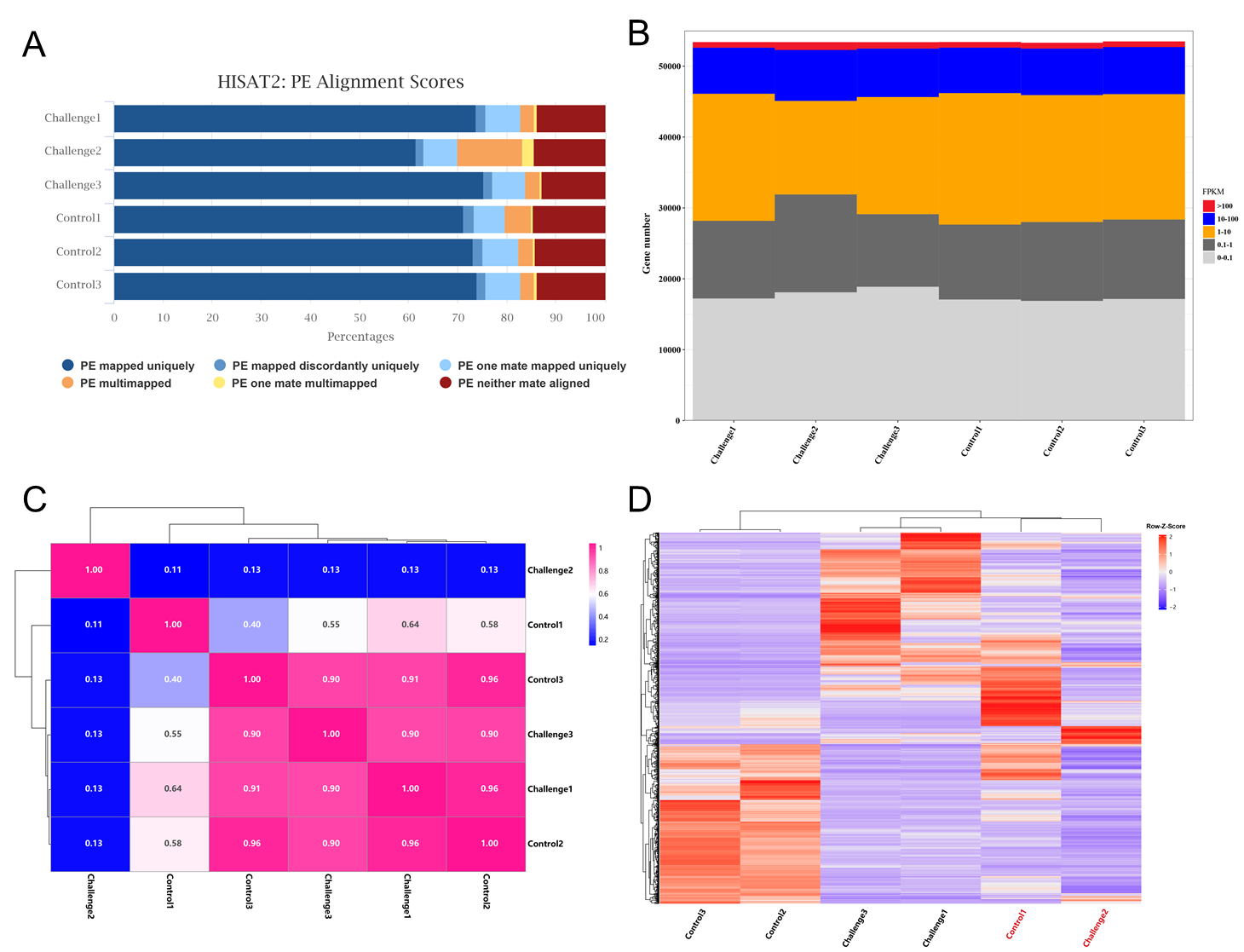


## Fig. S1.


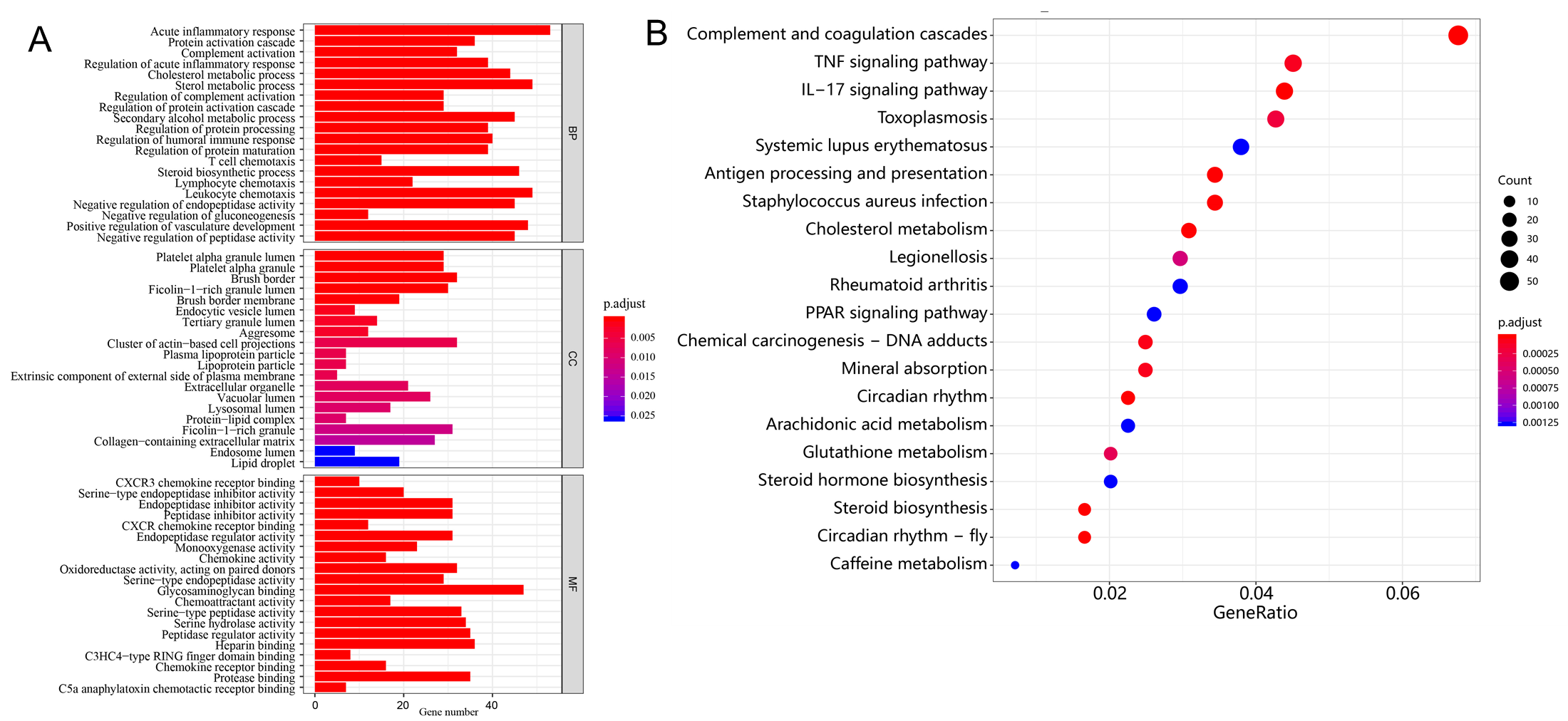


## Fig. S2.
